# Diabetes mellitus suppresseshemodialysis-induced increases in tear fluid secretion

**DOI:** 10.1186/1756-0500-7-78

**Published:** 2014-02-04

**Authors:** Motoko Nakata, Yuka Okada, Hirotsugu Kobata, Takashi Shigematsu, Peter S Reinach, Katsuo Tomoyose, Shizuya Saika

**Affiliations:** 1 Department of Ophthalmology, Kihoku Bun-in Hospital, Wakayama Medical University School of Medicine, 219 Myouji, Katsuragicho, Wakayama 649-7113, Itogun, Japan; 2 Department of Ophthalmology, Wakayama Medical University School of Medicine, 811-1 Kimiidera, Wakayama City 641-8509, Wakayama, Japan; 3 Nate Private Hospital, 294-1, Nateichiba, Kinokawa City 649-6631, Wakayama, Japan; 4 Department of Nephrology, Wakayama Medical University School of Medicine, 811-1, Kimiidera, Wakayama City 641-8509, Wakayama, Japan; 5 Biological Sciences, State University of New York, State College of Optometry, 33 West 42nd Street, New York, NY 10036, USA

**Keywords:** Tear, Hemodialysis, Diabetes mellitus

## Abstract

**Background:**

Hemodialysis is essential for the survival of patients suffering from chronic renal failure. However*,* in diabetics the incidence of dry eye disease is higher than in non-diabetic individuals. Accordingly, we evaluated if this difference is attributable to different effects of hemodialysis on basal tear fluid secretion.

**Methods:**

A modified Schirmer´s eye test determined if hemodialysis improved basal tear secretion rates in 36 diabetic and non-diabetic patients undergoing hemodialysis.

**Results:**

Basal tear secretion was invariant in diabetic patients whereas in non-diabetic individuals this process increased.

**Conclusion:**

In non-diabetic patients, autonomic neuropathy appears to be less severe and somewhat reversible since only in these individuals hemodialysis improved basal tear fluid secretion. This difference may be a factor contributing to the lower incidence of dry eye disease in non-diabetic patients.

## Background

Hemodialysis can be a life saving procedure for patients suffering from chronic renal failure. Nevertheless, its usage may lead to various complications, including dialysis disequilibrium syndrome [1], arteriovenous graft infection [2], cardiovascular disorders [3], autonomic nervous system dysfunction [4,5], psychiatric problems [6], and hyperparathyroidism [7]. Ophthalmic complications include visual disturbances [8], dysregulation of intraocular pressure [9-12], macular edema [13], calcium deposition in the cornea and conjunctiva [14], squamous metaplasia of the conjunctival epithelium [15], and decreased tear secretion [15-17]. Charlton et al. reported asymptomatic dry eye in dialyzed patients and noted that urea was present in the tear fluid [16]. Among patients in another study undergoing hemodialysis for chronic renal failure, the incidence of reduced basal tear secretion and dry eye symptomology is higher in diabetic patients than in non-diabetics [17]. Even though the incidence of dry eyes is higher in diabetic children suffering from chronic kidney failure, there are no reports on whether or not hemodialysis offsets declines in tear fluid secretion [18]. Similarly, in adult diabetic dry eye patients undergoing hemodialysis, it is unclear if this procedure increases tear fluid secretion [17,19]. To address this question, we determined in diabetic and non-diabetic patients suffering from chronic renal failure whether or not hemodialysis increases basal tear fluid secretion.

We show here that hemodialysis only increased basal tear secretion in non-diabetic patients. This difference suggests that in diabetic patients autonomic control of lacrimal gland function is irreversibly compromised by neuropathy whereas in non-diabetics the damage is less severe.

## Methods

### Subjects

Thirty-six chronic renal failure patients were enrolled in this study who received hemodialysis at the Nate private hospital (Wakayama, Japan). Seventy-one eyes were examined [26 male subjects (51 eyes) and 10 female subjects (20 eyes)]. Ages ranged from 42 to 87 (mean ± SD: 68.9 ± 10.2) years. Tables 1 and 2 provide their clinical signs that include ultrafiltration volume, blood urea nitrogen (BUN), serum creatinine levels, and duration of hemodialysis treatment.

**Table 1:** Data on non-diabetic mellitus patients afflicted with chronic renal failure

**Schirmer value before dialysis (mm)**	**0-36 (mean: 6.4 ± 7.8)**
Schirmer value after dialysis (mm)	1-24 (mean: 7.1 ± 4.5)*
Number of eyes	39 (male: 25, female: 14)
Age (yeas old)	42-86 (mean: 71.5 ± 10.6)
Ultrafiltration volume (ml/HD)	500-3,700 (mean: 2,231.0 ± 871.0)
BUN level (mg/dl)	48-92 (mean: 65.9 ± 12.3)
Serum creatinine level (mg/dl)	7.06-14.32 (mean: 10.4 ± 2.2)
Duration of hemodialysis treatment (months)	7-169 (mean: 49.2 ± 43.4)

*Wilcoxon signed-ranks test, p < 0.05.

**Table 2:** Data on diabetes mellitus patients afflicted with chronic renal failure

**Schirmer value before dialysis (mm)**	**1-16 (mean: 6.6 ± 3.9)**
Schirmer value after dialysis (mm)	0-17.5 (mean: 6.0 ± 3.9)
Number of eyes	32 (male:26 , female:6 )
Age (yeas old)	46-87 (mean: 65.8 ± 9.0)
Ultrafiltration volume (ml/HD)	1,100-3,400 (mean: 2,469.0 ± 680.0)
BUN level (mg/dl)	42-89 (mean: 67.5 ± 11.9)
Serum creatinine level (mg/dl)	4.47-13.94 (mean: 9.9 ± 2.5)
Duration of hemodialysis treatment (months)	3-97 (mean: 42.8 ± 30.8)

### Study design

The protocol of the study was approved by the Institutional Review Board at the Wakayama Medical University, Wakayama, Japan. Informed consent was obtained from all subjects. Tear secretion was evaluated by using the Schirmer´s test with topical anesthesia before and 5 min after hemodialysis. In this test, a strip of filter paper is applied between the lower palpebral conjunctiva and bulbar conjunctiva. The distance that fluid traverses down its length is measured after 5 min. Specifically, we used the Schirmer I test which is performed without topical anesthesia and the eyelids are left open permitting unrestricted blinking. The results are considered to represent the total retained and secreted tear fluid (basal and stimulated secretion volumes). To evaluate instead just basal tear secretion, topical anesthesia is applied, which eliminates stimulated secretion caused by filter paper application. Basal tear secretion was measured just before and immediately after hemodialysis.

Measurements were made in the eyes of 32 patients with diabetes mellitus and 39 others not diagnosed as diabetic. Statistical analysis was performed with Statcel2. Values are shown as means ± SD. Results were significant if values were p < 0.05. Wilcoxon signed-ranks test tested for significant changes between basal tear secretion values measured before and after hemodialysis. The Mann–Whitney U test determined if the changes were significantly different between the diabetic and non-diabetic groups.

## Results

Tables 1 and 2 provide clinical data in non-diabetic and diabetic patients respectively, and show basal tear secretion values before and after hemodialysis in these two patient groups. Basal tear secretion after hemodialysis was only larger than before performing this procedure in the non-diabetic group (Wilcoxon signed-ranks test, p < 0.05). On the other hand, there were no significant differences in serum creatinine level, dehydration volume, or duration of hemodialysis treatment between the diabetic and non-diabetic groups. In the non-diabetic group, their average age was older than those in the diabetic group (Mann–Whitney test, p < 0.01).

## Discussion

Autonomic innervation of the lacrimal gland is essential for controlling tear fluid secretion. Rises in urea resulting from chronic renal failure induce autonomic neuropathy leading to inappropriate control of lacrimal gland function. We found that in patients suffering from chronic renal failure that basal tear secretion was invariant in diabetic patients undergoing hemodialysis whereas it increased in the non-diabetic group. This difference suggests that the diabetic condition has an irreversible injurious effect on a neural component mediating control of lacrimal gland secretion.

Tears are an extremely complex mixture of numerous components including different types of mucin suspended in a fluid of body-like composition. The aqueous and proteinaceous components are secreted from the main and accessory lacrimal glands [20]. Meibomian gland secretion of hydrophobic meibum prevents excessive tear film evaporation and safeguards against increases in tear film osmolarity. Secretion of aqueous tears from the lacrimal glands is modulated by the trigeminal-parasympathetic nerve reflex. Sensory control is mediated by afferent sensory regulation and efferent parasympathetic and sympathetic regulation. In addition, the parasympathetic nerves are essential for regulation of aqueous tear secretion [21]. Diabetes mellitus correlates with both reduced corneal sensation and aqueous tear production [19]. Increases in mucin secretion by conjunctival goblet cells are dependent on elevated parasympathetic input to stimulate this response [22]. The failure of hemodialysis to stimulate basal tear secretion in diabetic patients suggests that this disease disrupts a component of autonomic neural input needed to sustain tear film formation and ocular surface health.

We employed the basal tear secretion test that requires topical anesthesia in order to eliminate reflex secretion and therefore evaluate autonomic nervous system-involved tear secretion. Our results regarding the higher prevalence of reduced tear secretion in diabetic than in non-diabetic patients undergoing dialysis patients agree with a previous report [17]. We show here for the first time that hemodialysis only increased basal tear fluid secretion in a non-diabetic group. The reason for this difference may stem from a consideration of the renal imbalance that hemodialysis only transiently mitigates. Commonly during chronic renal failure, uremia develops between dialysis sessions causing autonomic nerve impairment in chronic renal failure patients [4]. Uremia may be one of the major causative factors in this impairment [5]. For example, hypertension in chronic renal failure patients is considered to be due to autonomic sympathetic overactivity, but is not volume dependent [23]. Sympathetic hyperactivity is reportedly improved by short daily periods of hemodialysis [24].

The increases in basal tear secretion after dialysis may be a consequence of a decrease in uremia-induced autonomic neuropathy, as reported by Giordano et al. [25]. In contrast, in the diabetic group basal tear secretion did not increase even though autonomic neuropathy also occurs in these patients [26,27]. Despite this similarity, autonomic neuropathy improvement caused by hemodialysis appears to be less in the diabetic patients. In non-diabetic patients, sympathetic nerve activity was dominant before hemodialysis, but parasympathetic nerve activity became dominant after dialysis, whereas this change was not observed in diabetic patients. This difference may be due to autonomic neuropathy induction by uremia in the non-diabetic patients, whereas in the diabetic patients autonomic neuropathy is more severe and irreversible since it is induced by both uremia and metabolic dysfunction [25]. Accordingly, the invariance in basal tear fluid secretion in the diabetic patients is possibly ascribable to more extensive disruption of neuronal control of lacrimal gland function. This deficiency may account for the increase in dry eye incidence in these patients since the anterior ocular surface health may be compromised by being less hydrated than in non-diabetic patients.

## Conclusion

The increased incidence of dry eye disease in diabetic patients undergoing hemodialysis may be associated with the failure of this procedure to increase basal tear fluid secretion. Even though hemodialysis transiently decreases uremia irrespective of the diabetic condition and improves autonomic nerve function compromised by an elevated urea level, this procedure does not appear to reverse diabetic autonomic neuropathy.

## Competing interests

The authors declare that they have no competing interests.

## Authors’ contributions

MN and YO : Prepared the manuscript. SS and YO : contributed the study design. MN and HK: contributed data collection. MN, YO, TS, PR, TK, SS: were involved in the analysis and critical review of the manuscript. All authors read and are agreement with the final manuscript.
